# Unpredictable maternal sensory signals in caregiving behavior are associated with child effortful control

**DOI:** 10.1371/journal.pone.0279384

**Published:** 2022-12-20

**Authors:** Eeva Holmberg, Eeva-Leena Kataja, Elysia Poggi Davis, Marjukka Pajulo, Saara Nolvi, Hetti Lahtela, Elisabeth Nordenswan, Linnea Karlsson, Hasse Karlsson, Riikka Korja

**Affiliations:** 1 Department of Clinical Medicine, FinnBrain Birth Cohort Study, Turku Brain and Mind Center, University of Turku, Turku, Finland; 2 Department of Psychology and Speech-Language Pathology, University of Turku, Turku, Finland; 3 Department of Psychology, University of Denver, Denver, CO, United States of America; 4 Department of Pediatrics, University of California, Irvine, CA, United States of America; 5 Department of Child Psychiatry, University of Turku, Turku, Finland; 6 Department of Psychology and Speech-Language Pathology, Turku Institute for Advanced Studies, University of Turku, Turku, Finland; 7 Department of Psychiatry, University of Turku and Hospital District of Southwest Finland, Turku, Finland; 8 Centre for Population Health Research, University of Turku and Turku University Hospital, Turku, Finland; 9 Department of Pediatrics and Adolescent Medicine, University of Turku and Hospital District of Southwest Finland, Turku, Finland; Leiden University: Universiteit Leiden, NETHERLANDS

## Abstract

Emerging evidence suggests that exposure to unpredictable patterns of maternal sensory signals during infancy is associated with child neurodevelopment, including poorer effortful control. However, longitudinal effects on child development and possible sex differences are understudied. The aims of the present study were to explore whether exposure to unpredictable maternal sensory signals during infancy is related to child effortful control at 5 years of age and whether child sex moderates these associations. In addition, we examined how exposure to very high vs. low/moderate unpredictability using categorical cut-offs is related to child effortful control. Participants (133 mother–child pairs, all Caucasian) were drawn from the FinnBrain Birth Cohort Study in Finland. Maternal sensory signals (auditory, visual, tactile) were coded from the 10-min free-play episode on a moment-on-moment basis using Observer XT 11 (Noldus), and the unpredictability of maternal sensory signals was characterized as the entropy rate when the infant was 8 months of age. Child effortful control was assessed via mother reports using the Child Behavior Questionnaire very short form (CBQ-VSF) when the child was 5 years old. Correlational analyses showed that higher unpredictability of maternal sensory signals had a modest association with children’s poorer effortful control at 5 years of age. Notably, the linear regression model showed that child sex moderated these associations, as higher exposure to unpredictable maternal sensory signals was related to poorer effortful control among males, but not among females. Moreover, the general linear model showed that exposure to very high unpredictability was associated with poorer child effortful control at 5 years of age and remained significant when adjusted for possible confounding factors. These results are in line with previous findings and suggest that the unpredictability of maternal sensory signals is potentially an important aspect of early caregiving behavior associated with the development of child effortful control.

## Introduction

Early care is known to have profound effects on children’s later cognitive and emotional development [1–4]. The effects of early psychosocial deprivation [5,6] and insensitive parental caregiving behavior on children’s developing brains and stress regulation are well-documented [7–9]. In addition, parental emotional unpredictability and inconsistent parenting behaviors are known to contribute to children’s later psychosocial adjustment [10–12].

A novel study paradigm examining the unpredictability of maternal sensory signals in caregiving behavior conceptualizes unpredictability as the consistency of the sequences of maternal auditory, tactile, and visual signals during interactions with an infant [13]. The theoretical grounding for the model of unpredictability is based on the fundamental biological principles that neuronal circuits are shaped by patterns of sensory information, which has been documented most clearly in sensory systems, such as auditory and visual [14,15]. The current paradigm suggests that unpredictable patterns of sensory signals may be a relevant aspect of caregiving behavior that potentially adversely influences child brain maturation during sensitive periods of development, contributing to vulnerability to later cognitive and emotional disorders [15–18]. A previous cross-cultural study that included the present sample found associations between exposure to unpredictable maternal sensory signals and poorer child effortful control until 9.5 years of age in the US sample and until 2 years of age in the present (Finnish) sample [18]. However, the effects of exposure to unpredictable maternal sensory signals on the long-term development of child effortful control and possible sex differences have rarely been examined.

Patterns of sensory input early in life are required for the maturation of sensory circuits. For example, patterns of light are critical for the normative development of the visual system [19–21]. The impact of patterns of sensory information on cognitive and emotional systems is less well understood [15]. In both human and rodent research, the unpredictability of the sequences of the sensory signals (auditory, tactile and visual) that the mother provides during interactions with her child can be characterized by computing the entropy rate [13,17]. The entropy rate characterizes the predictability of the sensory signals a mother provides to her infant; the extent to which the current signal predicts what signal will occur next.

Experimental research with rodents has demonstrated, in simulated poverty experiments, that impoverished bedding conditions lead to unpredictable caregiving behavior by dams [22,23]. In turn, exposure to unpredictable care has been associated with offspring memory deficits and anhedonia [17,23–25]. In these experimental models, maternal genetic effects on offspring development were controlled via cross-fostering. Pups exposed to unpredictable care had worse cognitive and emotional outcomes compared to pups exposed to predictable care, and these findings have been replicated repeatedly [22].

Consistent with experimental research, recent human work has suggested that unpredictable patterns of maternal sensory signals while a mother interacts with her infant are associated with impairments in early childhood cognitive and emotional development, such as cognition, memory, effortful control, and cortisol responses [17,18,26]. Exposure to unpredictable maternal sensory signals in infancy has recently been associated with child neurocircuit development. Unpredictability of maternal sensory signals during infancy is associated with desynchronized maturation of corticolimbic pathways in children 9–11 years of age, which in turn associated with impairments of episodic memory [27].

Sex-specific responses to exposure to unpredictable sensory signals are still largely undiscovered. However, preliminary evidence from cross-species research shows that functions vulnerable to exposure to maternal unpredictable sensory signals may differ by sex [18,27,28]. Similarly, exposure to early-life stress is known to have sex-specific effects [29,30]. Moreover, it has been suggested that there may be some sex-differentiated pathways for how caregiving behaviors influence child development [31–34]. Studies have shown that males could be more vulnerable to the harmful effects of low caregiving quality in relation to developmental outcomes, such as externalizing symptoms and self-regulation difficulties [31,32,34]. However, the methodologies used vary widely, and a systematic approach is still needed to examine whether caregiving behaviors differentially influence the development of males and females.

One developmental outcome examined in relation to exposure to unpredictable maternal sensory signals is child self-regulation [18]. Self-regulation typically refers to goal-directed activities in the regulation of cognition, behavior, and emotion that are thought to develop in interaction with the early environment [35]. Moreover, self-regulation deficits are known to relate to later psychopathology [36,37], underscoring the importance of understanding which aspects of early care may contribute to self-regulation development. One aspect of self-regulation is effortful control (EC), which refers to the temperament-based capacity to flexibly control behavior and attention [38,39]. Emerging self-regulatory capacities can be noted in infants between 6 to 12 months, and these capacities develop rapidly during childhood [38] with females having higher EC beginning in early childhood compared to males [40]. According to the intergenerational transmission framework of self-regulation variables like socioeconomic factors, maternal effortful control, maternal mental health, and quality of care (i.e., maternal sensitivity) may contribute to child effortful control. These variables should thus be considered possible confounding factors when examining child effortful control [35].

In the present sample, it was previously found that the unpredictability of maternal sensory signals related to child EC until 2 years of age [18]. However, possible sex differences, as well as important possible maternal confounding factors such as maternal effortful control or maternal sensitivity, were not included in the previous study. In the present work, we addressed this gap in the literature by investigating whether exposure to unpredictable maternal sensory signals in infancy is associated with child EC at 5 years of age. Furthermore, given known sex differences both in responses to early-life experiences and in EC [29,31,32,40] we explore whether there are sex differences in these associations. In addition, we examine whether unpredictable sensory signals are associated with child effortful control after we covary possible confounding factors (i.e., maternal effortful control and maternal sensitivity). Previous work has evaluated unpredictable sensory signals (the entropy rate) as a continuous measure. We explored whether child outcomes differ between children exposed to very high levels of unpredictability and children exposed to low/moderate levels of unpredictability. Exploration of the categorical cut-offs is important to understand whether children exposed to high levels of unpredictability form a specific risk group in relation to child developmental outcomes. Furthermore, it is possible to start to explore whether we can also identify those mother–child pairs in the clinical contexts (i.e., who are the mother–child pairs at risk).

Our research questions were as follows:

1. How is the unpredictability of maternal sensory signals during a mother’s interactions with her infant at 8 months of infant age associated with child effortful control at 5 years of age?
  A previous cross-cultural study [18] found associations between exposure to more unpredictable maternal sensory signals in infancy and poorer child effortful control at 2 years of age in the present sample, and until 9.5 years of age in a US sample. Based on these previous findings, we hypothesize that unpredictable maternal sensory signals during a mother’s interactions with her infant will be negatively associated with child effortful control at the age of 5 years.
2. Are associations between unpredictability of maternal sensory signals at 8 months of infant age and child effortful control at 5 years of age moderated by child sex?
  Previous studies reported both sex-specific responses to early adversity and quality of caregiving behaviors, as well as sex differences in child effortful control [29,31,32,40]. Therefore, it is important to evaluate sex differences in response to unpredictability. Due to the lack of previous studies evaluating sex differences in response to unpredictability, we do not propose a specific hypothesis.
3. Exploratory question: Is very high vs. low/moderate unpredictability of maternal sensory signals at 8 months of age associated with poorer child effortful control at 5 years of age?
  We hypothesize that very high vs. low/moderate unpredictability is associated with poorer child effortful control at 5 years of age.

## Methods

### Study design and participants

The present study sample (n = 133) was drawn from the FinnBrain Birth Cohort Study (n = 3808) [41]. The main aim of the FinnBrain study is to prospectively explore the effects of prenatal and early-life stress on child health and development. Recruitment took place during the first trimester free-of-charge ultrasound (gestational week [GW] 12) by a research nurse between December 2011 and April 2015 in the South-Western Hospital District and Åland Islands in Finland. A written informed consent was provided by the parents prior to the study visits for children. Information on maternal age and infant biological sex at birth was obtained from the national birth registries (data from national birth registries, National Institute for Health and Welfare, www.thl.fi) and data was fully anonymized before access to the registries. The Ethics Committee of the Hospital District of Southwest Finland approved the study protocol.

A nested case-control population, i.e., a focus cohort, was drawn from the main cohort to examine in more detail the effects of prenatal stress exposure on child development. The criteria for the focus cohort were determined by using the questionnaire data of the first 500 participant mothers’ to establish cut-points for the approximately highest and lowest 25th percentiles of maternal depressive and anxiety symptoms during pregnancy. More detailed criteria for the focus cohort are described in Karlsson et.al [41]. Focus cohort families were invited to a Child Development and Parental Functioning Lab study visit when their infants were 8 months of age. The study visit included an assessment of mother–infant interactions.

### Measures

#### Unpredictability of maternal sensory signals (i.e., the entropy rate)

Maternal sensory signals (auditory, visual and tactile) were coded from 10-min free-play episodes on a moment-on-moment basis using the Observer XT 11 (Noldus). Auditory signals included all maternal vocalizations (e.g., speech and laughter). Visual signals included maternal manipulations of a toy while the infant was visually attending. Tactile signals involved all physical contact initiated by the mother (e.g., touching and holding). Inter-rater agreement was calculated for 10% of the videotapes and averaged 86% between the independent coders.

Quantification of the unpredictability of maternal sensory signals was calculated with conditional probabilities of transitioning between different combinations of maternal auditory, visual, and tactile sensory signals, considering all eight possible combinations of these signals (presence/absence of input of each of the three types of sensory signals). For example, a mother who is holding her infant and talking to her is providing tactile and auditory sensory input to the infant simultaneously. If the mother additionally shows a toy to the infant, she is now providing tactile, auditory, and visual sensory input simultaneously. This is considered a transition between different combinations of signals (from a combination of tactile and auditory signals to a combination to all types of signals).

The transitions between different combinations of maternal sensory signals are modeled as changes in the state of a discrete-state first-order Markov process, and the entropy rate of the process determines the unpredictability of maternal sensory signals. If one combination of sensory signals (e.g., tactile and auditory signals simultaneously) always follows a specific combination (e.g., all signals), that is considered predictable. In contrast, if the transition from one combination of sensory signals to the next is random, it is considered unpredictable. The entropy rate ranges from 0 to 2.807, with higher values indicating higher unpredictability in transitions between different combinations of sensory signals. Materials regarding the calculation of the entropy rate are provided in Davis et al. [17] and available at https://contecenter.uci.edu/shared-resources/. The number of transitions did not correlate with the entropy rate (r = -.003, p = .972), indicating that the entropy rate is a separate construct from the number of transitions.

The unpredictability of maternal sensory signals (i.e., the entropy rate) was used as a continuous and a categorical variable in the analyses. The cut-offs for the entropy rate were derived from another interaction measurement (i.e., maternal sensitivity) used in the present study. Theoretically based and empirically validated cut-off for “low sensitivity” (scores 1 to 3.5) [42] formed 15% in the present sample, and similarly, cut-offs for the entropy rate were set up to the highest 15th percentile (entropy rate: 1.080–1.348) and lowest 85th percentile (entropy rate: 0.412–1.076) to create a similar extreme group.

### Child effortful control

Child effortful control was assessed with mother reports using the Child Behavior Questionnaire very short form (CBQ-VSF) [43] when the child was 5 years of age. The CBQ-VSF includes 36 items forming three factors: effortful control, negative affectivity, and surgency. The effortful control factor, consisting of 12 items (e.g., “When drawing or coloring in a book, shows strong concentration” or “Is good at following instructions”), was used in the present study. The CBQ is a valid and reliable questionnaire for assessing child reactivity and regulation in children between 3 and 7 years [43,44]. Mothers rate their children’s observed behavior during the previous 6 months on a scale from 1 to 7. A higher score for each item or factor reflects a higher level of a particular behavior. The effortful control factor showed adequate internal consistency in the present study (α = 0.76).

#### Confounding factors

According to the theories describing the intergenerational transmission of self-regulation and previous empirical studies, factors such as socioeconomic status, maternal effortful control, maternal mental health, and quality of care (i.e., maternal sensitivity) may contribute to both parental caregiving and child effortful control [35,45–47]. Thus, these factors, more specifically economic satisfaction, maternal effortful control measured when the child is 1 year old, maternal anxiety and depressive symptoms, and maternal sensitivity were considered as potential confounders.

#### Socioeconomic status

Economic satisfaction was used to control for socioeconomic status, while education was omitted from the models because it was related to child effortful control and maternal unpredictability in an unexpected (nonlinear) manner that should be further explored. Economic satisfaction was measured with a visual analogue scale on which the person could draw a mark on the line where they felt their satisfaction was. The scale ranges from 0 to 10, with a higher value indicating higher economic satisfaction.

#### Maternal sensitivity

Maternal sensitivity was assessed from a 20-min video recordings using the Emotional Availability (EA) Scales [48]. The EA Scales are rooted in attachment theory but expand it by emphasizing the importance of emotional expression in the adult–child dyad [9]. The EA Scales consist of scales of maternal sensitivity, structuring, non-intrusiveness and non-hostility, and child responsiveness and child involvement. In the present study, we used only the scale for maternal sensitivity. Maternal sensitivity refers to a mother’s ability to recognize her child’s interaction cues and respond to them appropriately and in a timely manner [48]. Maternal sensitivity is evaluated from 1 to 7, where a higher score indicates higher sensitivity. Scores of 1 to 2 are considered problematic/disturbed interaction, and scores of 2.5 to 3.5 refer to detachment in the interaction. Scores of 4 to 5 are considered somewhat problematic/complicated interaction, and scores of 5.5 to 7 refer to emotionally available interactions between the adult and child [9,42,48]. Maternal sensitivity was used as a continuous and a categorical variable in the present study. In the present sample, 15% of the mothers scored below 3.5, which is considered the “low sensitivity group”. Inter-rater reliability was assessed for 10% of the videotapes, and the intraclass correlation coefficient was 0.80 for maternal sensitivity.

#### Maternal effortful control

Maternal effortful control was assessed with self-reports using the Adult Temperament Questionnaire (ATQ) [49] when the child was 1 year of age. The ATQ includes 77 questions consisting of factors of effortful control, negative affect, extraversion and orienting sensitivity. The factor effortful control, consisting of 19 items (e.g., "I can keep performing a task even when I would rather not do it” or “When interrupted or distracted, I usually can easily shift my attention back to whatever I was doing before”), was used in the present study. The effortful control factor showed good internal consistency (α = .83).

#### Maternal anxiety and depressive symptoms

Maternal anxiety symptoms were assessed with self-reports using the Symptom Checklist (SCL-90) [50,51], and maternal depressive symptoms were assessed with self-reports using the Edinburg Postnatal Depression Scale (EPDS) [52] when the child was 5 years of age. The SCL-90 consists of 10 items rated from 0 to 4, and the EPDS of 10 items rated from 0 to 3. Both measures showed good internal consistency (α = .89 for both the SCL-90 and the EPDS).

### Procedure

Mothers filled out a background information questionnaire at GW 14, including their education level, monthly income, economic satisfaction, and parity. Information on maternal age and infant biological sex at birth was obtained from the national birth registries (data from national birth registries, National Institute for Health and Welfare, www.thl.fi). Mother–child interaction was assessed in a video-recorded free-play situation when the infant was 8 months of age. The mother was given a standard set of toys and asked to play for 20 min together with her infant as they would play at home. The video-recorded free-play situations were analyzed with two different coding methods: unpredictability of maternal sensory signals and the EA Scales (see measures above). Maternal effortful control was assessed with a self-report questionnaire when the child was 1 year of age, and maternal anxiety and depressive symptoms were assessed with a self-report when the child was 5 years of age. Finally, child effortful control was assessed with a mother report when the child was 5 years of age.

### Data analysis

SPSS 28.0 was used for the statistical analyses. The entropy rate and child effortful control showed normal distributions. Missing data (< 10%) for maternal anxiety (the SCL-90) and depressive (the EPDS) symptoms at 5 years of child age were imputed using the random forest method (MissForest) [53] in R 3.6.1. Missing data for maternal effortful control (20%) and background information (i.e., education, income, economic satisfaction, and parity; 1%) were imputed using multiple imputation (10 imputations) using the MCMC protocol. According to Little’s MCAR test, data were missing at random (χ^2^(2) = 4.949, p = .084).

Associations between possible confounding factors and child effortful control were examined using Pearson correlations and a t-test. Confounding factors yielding at least a small effect size (r >.10) or significant association with the outcome were included as covariates. Associations between the unpredictability of maternal sensory signals and child effortful control were assessed using Pearson correlations. Linear regression (stepwise) was conducted to see whether the associations remain significant after adjustment for economic satisfaction (step 1), maternal effortful control (step 2), and maternal sensitivity and child sex (step 3).

The interaction effect between unpredictable maternal sensory signals and child sex was explored with a standard linear regression. In the first step, covariates (economic satisfaction, maternal effortful control, and maternal sensitivity) were added to the model. In the second step, the main effects of unpredictability and child sex were entered in the model. Finally, the interaction term between unpredictability and child sex was entered in the model. The interaction effect was additionally explored using Pearson correlations.

Group comparisons using categorical cut-offs for unpredictability were assessed using a t-test. Next, a general linear model was used to test whether the association remained when significant covariates (economic satisfaction, maternal effortful control, categorical maternal sensitivity, and child sex) were included. Finally, the interaction effect between categorical unpredictability and child sex was tested.

#### Preliminary analysis

The associations between confounding factors and child effortful control are shown in Table 1. Maternal economic satisfaction (r = .126, p = .151), maternal effortful control (r = .136, p = 160), and maternal sensitivity (r = .128, p = .141) had modest positive associations (r > .10) with child effortful control and thus, were included in the additional analyses as covariates. Child sex was significantly related to effortful control, with males displaying poorer effortful control compared to females (t (131) = -3.569, p < .001, d = .62), and was included in the models as well. Maternal anxiety (r = -.054, p = .540) and depressive symptoms (r = -.058, p = .511) at 5 years of child age did not meet the criteria for inclusion as a covariate and thus were excluded from the following models.

**Table 1:** Pearson correlations between possible confounding factors, maternal unpredictable sensory signals, and child effortful control.

	Child effortful control
Maternal unpredictable sensory signals	-.172*
Economic satisfaction	.126
Maternal effortful control	.136
Maternal sensitivity	.128
Maternal anxiety symptoms (5 y)	-.054
Maternal depressive symptoms (5 y)	-.058

* p < .05.

## Results

### Sample characteristics and attrition

For the 8-month mother–child interaction assessment, 354 families were invited, and 195 of them participated (55.1%). Non-participating mothers had lower education levels (χ^2^(2) = 14.07, p < 0.001) and were younger (t (313) = 2.35, p = 0.020, d = .25, CI = .044 - .465) than the participating mothers. Of the mother–infant interaction video recordings 180, were able to be coded. Videos were excluded if the father was in the video instead of the mother or if the video was too short (less than 7 min). Of these participants, 133 had mother reports for child effortful control at 5 years of age, filled out at home online (n = 109) or during neuropsychological study visits for 5-year-old children (n = 24). Mothers who did not complete the child self-regulation questionnaire at home online had lower education levels (χ^2^(2) = 103.89, p < .001), had lower monthly incomes (χ^2^(3) = 23.37, p < .001), and were younger (t (3268) = 8.304, p < .001, d = -.269, CI = -.335 - -.203) than mothers who completed the questionnaire. For the 5-year-old study visit, 1288 families were recruited, and 545 (42.3%) of them participated. Similarly, non-participating mothers had lower education levels (χ^2^(2) = 30.94, p < .001), had lower monthly incomes (χ^2^(3) = 11.65, p = .009) and were younger (t (1286) = -4.130, p < .001, d = -.233, CI = -.344 - -.122) than the participating mothers. The sample demographics are presented in Table 2.

**Table 2:** Demographic characteristics of the sample (n = 133).

		*Theoretical range*
Maternal education (%)		
High school/secondary education	20.5	
Polytechnical diploma	39.4	
University degree	40.2	
Monthly income (%) *		
1500 or less	32.6	
1501–2500	56.8	
2501–3500	9.1	
>3500	1.5	
Economic satisfaction, mean	6.15 (2.31)	0–10
Maternal age at delivery (years), mean	31.42 (3.86)	
Primiparous (%)	58.3	
Maternal effortful control, ATQ (child 1 y), mean	4.64 (.76)	1–7
Maternal sensitivity, EA (child 8 mo), mean	5.34 (1.40)	1–7
Maternal anxiety symptoms, SCL-90 (child 5 y), mean	4.63 (5.81)	0–40
Maternal depressive symptoms, EPDS (child 5 y), mean	5.19 (4.97)	0–30
Child sex (% female)	47.4	
Unpredictability of maternal sensory signals (child 8 mo), mean	.89 (.17)	0–2.807
Males mean	.91 (.16)	
Females mean	.88 (.19)	
Child effortful control, CBQ (5y), mean	5.46 (.74)	1–7
Males mean	5.25 (.75)	
Females mean	5.69 (.66)	

ATQ = Adult Temperament Questionnaire; EA = Emotional Availability Scale; SCL-90 = Symptom Checklist 90/anxiety subscale; EPDS = Edinburgh Postnatal Depression Scale; CBQ = Child Behavior Questionnaire.

* In Euros.

### Unpredictability of maternal sensory signals at 8 months of infant age and child effortful control at 5 years of age

Higher unpredictability of maternal sensory signals correlated with lower child effortful control at 5 years of age (r = -.172, p = .048, CI = -.333 - -.002); see Table 1.

In the adjusted models (see Table 3) association between unpredictability and child effortful control, remained at a trend-level after adjustments for economic satisfaction (β = -.167, p = .052, CI = -1.419 - .005). The associations weakened after adjustments for economic satisfaction and maternal effortful control (β = -.156, p = .077, CI = -1.389 - .072), and diminished after adjusting for economic satisfaction, effortful control, maternal sensitivity and child sex (β = -.118, p = .175, CI = -1.219 - .221). Child sex was also a significant predictor of child effortful control (β = .275, p = .001, CI = .161 - .652).

**Table 3:** Standard linear regression for child effortful control.

	Standardized β	Unstandardized β	p	95% CI	R^2^ (adj)
Step 1					.029
Maternal unpredictable sensory signals	-.167	-.707	.052	-1.419-.005	
Economic satisfaction	.119	.038	.167	-.016-.092	
Step 2					.027
Maternal unpredictable sensory signals	-.156	-.659	.077	-1.389-.072	
Economic satisfaction	.090	.029	.344	-.031-.089	
Maternal effortful control (ATQ)	.071	.070	.501	-.134-.274	
Step 3					.095
Maternal unpredictable sensory signals	-.118	-.499	.175	-1.219–221	
Economic satisfaction	.083	.026	.369	-.031-.084	
Maternal effortful control (ATQ)	.019	.019	.848	-.176-.214	
Maternal sensitivity (EA)	.103	.055	.228	-.034-.143	
Child sex (male)	.275	.406	.001	.161-.652	

ATQ = Adult Temperament Questionnaire; EA = Emotional Availability Scale.

### Moderation by child sex

**Fig 1:**
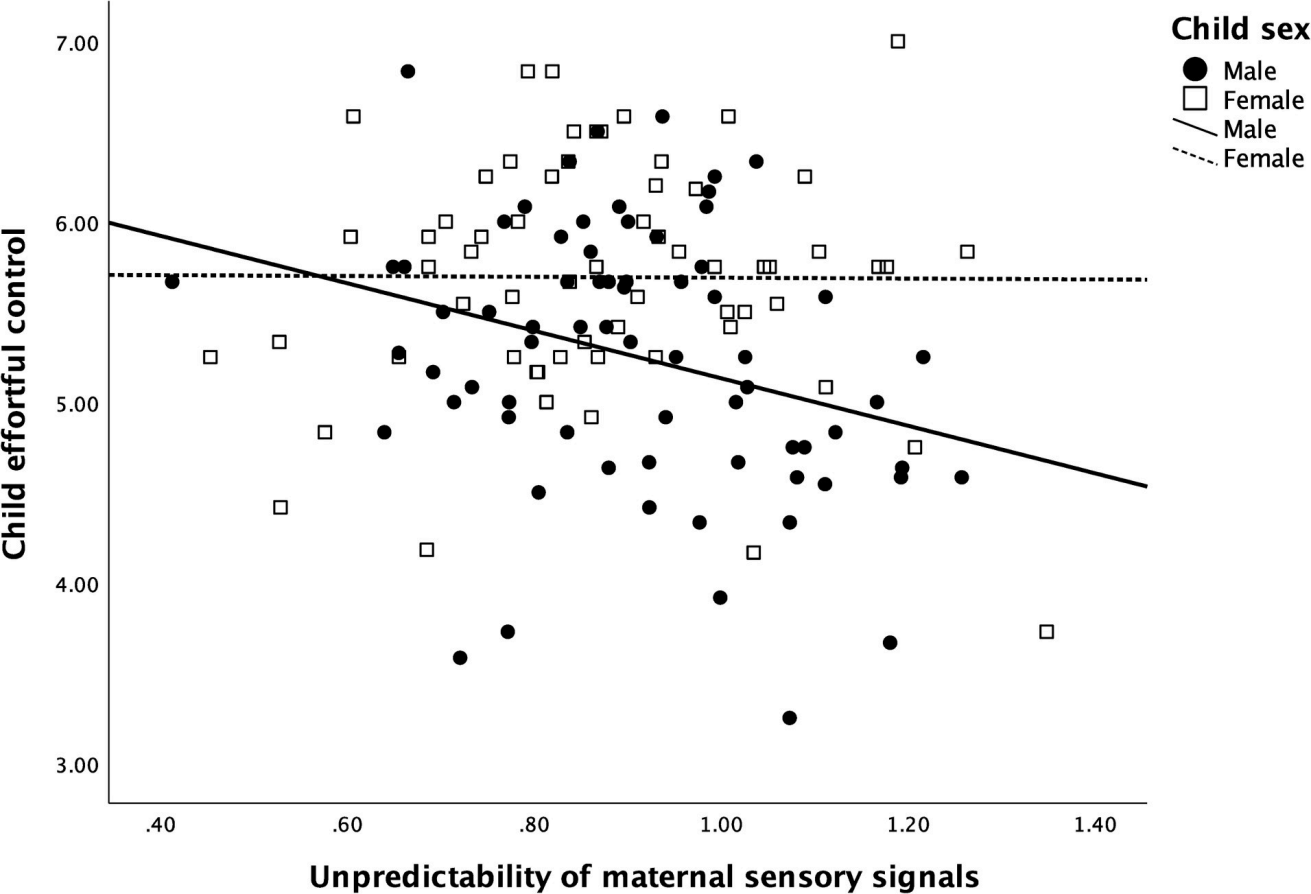
For males, exposure to unpredictable maternal sensory signals is related to effortful control at 5 years of age.

The models for moderation by child sex analysis are shown in Table 4. The test of the moderating role of child sex suggested that unpredictable sensory signals were differently related to child effortful control among male and female infants, although the association did not reach standard statistical significance when adjusted for economic satisfaction, maternal effortful control, and maternal sensitivity (β = .902, p = .062, CI = -0.66–2.687; see model step 3). Thus, follow-up analyses were used to probe these interactions. Probing revealed negative correlations between infant exposure to unpredictable maternal sensory signals and child effortful control at 5 years of age among males (r = -.289, p = .015, CI = -.491 - -.058), but not among females (r = -.007, p = .959 CI = -.254 - .242); see Fig 1.

**Table 4:** Standard linear regression for child effortful control.

	Standardized β	Unstandardized β	p	95% CI	R^2^ (adj)
Step 1					.018
Economic satisfaction	.092	.029	.338	-.031-.089	
Maternal effortful control (ATQ)	.087	.085	.404	-.116-.286	
Maternal sensitivity (EA)	.120	.064	.166	-.027-.154	
Step 2					.096
Economic satisfaction	.083	.026	.369	-.031-.084	
Maternal effortful control (ATQ)	.019	.019	.848	-.176-.214	
Maternal sensitivity (EA)	.103	.055	.228	-.034-.143	
Maternal unpredictable sensory signals	-.118	-.499	.175	-1.219–221	
Child sex (male)	.275	.406	.001	.161-.652	
	0^a^				
Step 3					.112
Economic satisfaction	.071	.023	.439	-.035-.080	
Maternal effortful control (ATQ)	.031	.023	.812	-.168-.214	
Maternal sensitivity (EA)	.113	.060	.183	-.028-.148	
Maternal unpredictable sensory signals	-.591	-2.499	.027	-4.718- -.280	
Child sex (male)	-.517	-.764	.232	-2.017-.489	
	0^a^				
Maternal unpredictable sensory signals by child sex	.902	1.311	.062	-0.66–2.687	

ATQ = Adult Temperament Questionnaire; EA = Emotional Availability Scale.

### Categorical variables

Infant exposure to high unpredictability of maternal sensory signals (highest 15th percentile) was significantly related to child poorer effortful control, M (SD) = 5.10 (.82), at 5 years of age (t(131) = -2.413, p = .016, d = -.59, CI = -1.065 - -.104), compared to low/moderate unpredictability of maternal sensory signals (lowest 85th percentile), M (SD) = 5.53 (.71).

The models testing this association when adjusted for covariates (economic satisfaction, maternal effortful control, categorical maternal sensitivity, and child sex) are shown in Table 5. High unpredictability of maternal sensory signals (the highest 15th percentile) remained significantly associated with poorer child effortful control at 5 years of age (β = .169 p = .047, CI = -.694 - -.004) even after the inclusion of covariates. Moreover, low maternal sensitivity (β = .175 p = .035, CI = -.696- -.025) and child sex (male) (β = .276 p < .001, CI = -.647- -.167) related significantly to poorer child effortful control as well (see Table 5).

**Table 5:** The general linear model for child effortful control (using categorical cut-offs).

	Standardized β	Unstandardized β	p	95% CI
Economic satisfaction	.070	.022	.439	-.034-.079
Maternal effortful control (ATQ)	.014	-.004	.968	-.195-.187
Maternal sensitivity (EA)				
Lowest 15th percentile	.175	-.360	.035	-.696- -.025
Highest 15th percentile	0^a^			
Maternal unpredictable sensory signals				
Highest 15th percentile	.169	-.349	.047	-.694- -.004
Lowest 85th percentile	0^a^			
Child sex (male)	.276	-.407	.001	-.647- -.167

ATQ = Adult Temperament Questionnaire; EA = Emotional Availability Scale.

Males exposed to high levels of unpredictable sensory signals in infancy (n = 11) had poorer effortful control M(SD) = 4.73 (.48) in comparison to the low/moderate unpredictability group (n = 59) M(SD) = 5.35(.75). Similarly, females (n = 9) exposed to high levels of unpredictable sensory signals in infancy had poorer effortful control M(SD) = 5.55 (.94) in comparison to the low/moderate unpredictability group (n = 54) M(SD) = 5.72 (.62). However, there was no significant interaction effect between categorical unpredictability and child sex in predicting child effortful control.

## Discussion

In the present study, we explored the associations between the unpredictability of maternal sensory signals in caregiving behavior in infancy and child effortful control at 5 years of age after adjustment for the effects of possible confounding factors (i.e., economic satisfaction, maternal effortful control, maternal sensitivity, and child sex). In addition, we explored whether very high vs. low/moderate unpredictability of maternal sensory signals at 8 months of age predicted child effortful control at 5 years of age. Furthermore, we tested whether there were any sex-specific responses to exposure to the unpredictability of maternal sensory signals. We found a modest association between higher unpredictability of maternal sensory signals in infancy and poorer child effortful control at 5 years of age. However, these associations weakened after adjustment for economic satisfaction and maternal effortful control and diminished after further adjustment for maternal sensitivity and child sex. Additionally, we found some preliminary evidence that exposure to high vs. low/moderate unpredictability of maternal sensory signals may play a specific role in child development. High vs. low/moderate unpredictability was significantly related to poorer child effortful control at 5 years of age even after adjustment of the significant covariates (economic satisfaction, maternal effortful control, maternal sensitivity, and child sex). Moreover, we found a trend-level finding for the moderating role of child sex: For males, infant exposure to unpredictable maternal sensory signals (using a continuous variable) was related to poorer effortful control at 5 years of age, whereas there were no significant associations in females.

This finding that exposure to unpredictable maternal sensory signals, especially at very high levels, is associated with children’s poorer effortful control is in line with findings in a previous study [18]. Previously, it has been shown that infant exposure to more unpredictable maternal sensory signals is related to lower emerging self-regulation capacities of the infant at 1 year of age in a US cohort and the present Finnish sample. Furthermore, longitudinal analyses indicated that these associations persisted until 9.5 years of age in the US sample and until 2 years of age in the Finnish sample used in the present study [18]. The present results showed a modest longitudinal association between higher unpredictability of maternal sensory signals and poorer child effortful control until 5 years of age. However, the association diminished in adjusted models with possible confounding factors (i.e., economic satisfaction, maternal effortful control, maternal sensitivity, and child sex), showing that several aspects affect the development of child effortful control capacity in early childhood. It is the case that unpredictable signals may be one of the pathways by which economic and social experiences shape the developing infant, which could be explored in future studies.

However, exposure to very high levels of unpredictability was associated with poorer child effortful control at 5 years of age even after all the possible confounding factors (i.e., economic satisfaction, maternal effortful control, maternal sensitivity, and child sex) were included. This finding indicates that the association between maternal unpredictable sensory signals and child outcomes may not be linear; rather, very high levels (the highest 15% percentile) of unpredictability may be more harmful for child brain maturation in comparison to exposure to moderate or low levels of unpredictable sensory signals. However, this analysis was exploratory, and more studies are needed in the future to validate and adjust the clinical cut-offs. This is especially important so that the unpredictability phenomenon could be considered in the clinical context. It would be important to understand the risk factors for the high unpredictability of sensory signals and to develop early screening tools for identifying these mother–child pairs at risk, as entropy measurement itself is not a clinically feasible tool. There is still little understanding of the maternal risk factors related to the high unpredictability of maternal sensory signals. However, previous research has shown that cumulative risk factors such as low maternal self-regulation together with high anxiety symptoms are associated with high unpredictability of maternal sensory signals [54], opening up one possible avenue for identifying mothers at risk in the clinical context. Moreover, the Questionnaire of Unpredictability in Childhood (QUICK) [12] was recently developed and validated to assess unpredictable parenting and home environments before the age of 18 years. However, more knowledge is still needed to understand how maternal unpredictable sensory signals can be defined and measured in the clinical setting and in daily parenting.

In addition, we found that children exposed to low maternal sensitivity (lowest 15th percentile) associated with poorer child effortful control. Together, these findings suggest that children in high-risk groups of non-optimal caregiving behavior may be most vulnerable to the adverse effects of low sensitivity and high unpredictability. Moreover, these results also highlight that unpredictable maternal sensory signals and maternal sensitivity seem to be clearly separate aspects of early caregiving behavior [17,55] and may have independent associations with child self-regulation.

Interestingly, we also observed possible sex differences in the associations between the unpredictability of maternal sensory signals and child effortful control. A trend-level finding indicated that exposure to unpredictable maternal sensory signals was related to poorer child effortful control among males, but not among females. There are several possible explanations for these findings. First, there are some innate differences in neural functioning between males and females, which could underlie early behavioral differences between males and females [56]. Second, exposure to early adversities has shown sex-specific effects [29,30]. Third, caregiving quality may have sex-differentiated pathways influencing child development, frequently showing that males are more vulnerable to the effects of low caregiving quality [31,32,34]. Finally, males are known to have a lower effortful control capacity from early childhood on compared to females [40]. Interestingly, this study indicates that male and female infants may respond differently to maternal unpredictable sensory signals in terms of their effortful control development, and that males may be especially susceptible to such environmental exposure. However, the findings are very preliminary, as the interaction effect remained at a trend-level only, after accounting for several covariates. Thus, these findings regarding possible sex differences should be replicated and further examined in the future.

This study is among the first to explore the effects of the unpredictability of maternal sensory signals on child development across early childhood. Decades of research evidence, based on the foundational work of Bowlby, have shown that the quality of early caregiving behavior is crucial for child cognitive and emotional development [3,7]. The present study paradigm adds a novel parameter to the nature of maternal caregiving behavior, i.e., patterns of maternal sensory signals that may be an additional contributor to child development. This is especially relevant, as sensory input from the environment during sensitive periods is known to be essential for maturation of sensory circuits [19–21], and it is possible that unpredictable sensory input early in life may disrupt the maturation of brain circuits involved in complex behavior as well [16,27,57]. Recent human research has suggested that an underlying mechanism explaining poor cognitive functions in children exposed to unpredictable maternal sensory signals could be the desynchronized maturation of corticolimbic pathways [27]. The present results are in line with this evolving knowledge by suggesting that child self-regulatory capacities may also be affected by exposure to unpredictable maternal sensory signals early in life. However, given the paucity of research on this topic, replications and further studies are needed to understand which areas in child development are most vulnerable to unpredictable maternal sensory signals in early life.

The present study has several strengths. Unpredictable maternal sensory signals were observed using a novel method with high inter-rater agreements [13]. Moreover, we were able to consider several relevant confounding factors in the associations between the unpredictability of maternal sensory signals and child effortful control, such as the effects of maternal effortful control, and maternal sensitivity. However, there are also some limitations to consider. The participating mothers had a high socioeconomic status, which limits generalizability. In addition, the results are specific to the cultural context and not necessarily generalizable to other countries. Genetic influence for mother or child behavior was not possible to control for. Furthermore, child effortful control was assessed only with mother reports, which might increase the risk of reporter bias. This concern was mitigated by showing that maternal mental health (i.e., current anxiety or depressive symptoms) was not associated with the mother reports of child effortful control. However, the CBQ is designed to characterize how a child responds in specific situations, rather than subjective ratings of the child. In addition, the sample size was somewhat small, and future studies should replicate these findings in larger samples.

To conclude, this study replicates the previous finding [18] that exposure to unpredictable maternal sensory signals during infancy is associated with child self-regulatory capacities (i.e., effortful control), and adds knowledge that males may be more susceptible to such exposure in terms of effortful control development. Preliminary analysis also indicated that the effect on child effortful control was stronger when a high vs. low/moderate level of unpredictability was considered, a finding that opens up new avenues in the research on unpredictability and its clinical implications (i.e., who are the children at risk, and what is an adequate level of caregiving unpredictability). These associations and possible sex differences should be investigated further to obtain a more comprehensive picture of this novel study paradigm and its significance for child development.
